# Pulmonary Embolism and ABO Blood Type: A Systematic Review 

**DOI:** 10.3390/diagnostics15232973

**Published:** 2025-11-24

**Authors:** Abdulkader Jamal Eddin, Oana Elena Tunea, Ioana Monica Mozos, Arnaldo Dario Damian, Stefan-Iulian Stanciugelu

**Affiliations:** 1 Department of Pathophysiology, Doctoral School of “Victor Babeş” University of Medicine and Pharmacy, 300041 Timişoara, Romania; jamal_eddin6@yahoo.com; 2 Center for Translational Research and Systems Medicine, “Victor Babeş” University of Medicine and Pharmacy, 300173 Timişoara, Romania; ioanamozos@umft.ro; 3 Gastroenterology and Hepatology Clinic, Pius Brînzeu County Emergency Clinical Hospital, 300723 Timișoara, Romania; 4 Advanced Research Center in Cardiovascular Pathology and Hemostaseology, “Victor Babeş” University of Medicine and Pharmacy, 300041 Timișoara, Romania; 5 Internal Medicine Clinic, Timisoara Municipal Emergency Clinical Hospital, 300254 Timișoara, Romania; 6 Department of Functional Sciences-Pathophysiology, “Victor Babeş” University of Medicine and Pharmacy, 300173 Timişoara, Romania; 7 Neurology Clinic II, Pius Brînzeu County Emergency Clinical Hospital, 300723 Timișoara, Romania; dariodamian996@gmail.com; 8 Orthopedics II Research Center, Pius Brînzeu County Emergency Clinical Hospital, 300723 Timișoara, Romania; stefan.stanciugelu@umft.ro; 9 Orthopedics Clinic II, Pius Brînzeu County Emergency Clinical Hospital, 300723 Timișoara, Romania

**Keywords:** pulmonary embolism, ABO blood group, venous thromboembolism, genotype, risk factor

## Abstract

**Background:** Pulmonary embolism (PE) remains a major cause of cardiovascular morbidity and mortality. Classical risk factors explain only part of the interindividual variability in thrombotic risk. Non-O blood groups are associated with higher plasma levels of von Willebrand factor and factor VIII, suggesting a potential prothrombotic mechanism. This systematic review and limited meta-analysis examined the relationship between ABO blood group and PE risk. **Methods:** Following PRISMA 2020 guidelines, PubMed, Embase, and Web of Science were searched through August 2025 for observational studies reporting ABO blood group and objectively confirmed PE in adults. Eligible designs included cohort, case–control, and registry-based studies. Two reviewers independently extracted data and assessed risk of bias using the Newcastle–Ottawa Scale. Comparable effect estimates were pooled using a random-effects model (DerSimonian–Laird method, inverse-variance weighting). **Results:** Four studies met inclusion criteria, comprising more than 300,000 participants from North America, Europe, and Asia. All reported a higher incidence of PE among non-O compared with O blood groups. Pooled analysis of two large population-based cohorts yielded a summary relative risk of 1.36 (95% CI 1.20–1.54; I^2^ = 2.6%), indicating a modest but consistent association. Data on recurrence, severity, and mortality were limited and heterogeneous. **Conclusions:** Non-O blood groups are associated with an approximately 30–40% higher risk of PE across diverse populations. While evidence is insufficient for causal inference, ABO phenotype represents a biologically plausible and readily available marker that may complement multifactorial models of thromboembolic risk.

## 1. Introduction

Pulmonary embolism (PE) ranks third among acute cardiovascular syndromes after myocardial infarction and stroke [1,2,3,4]. Its annual incidence in Europe and North America ranges from 39 to 115 per 100,000 individuals, and mortality remains 7–11% despite diagnostic and therapeutic progress [4,5,6]. Beyond the acute phase, PE contributes to chronic thromboembolic pulmonary hypertension (CTEPH), impaired quality of life, and substantial healthcare burden [3,6,7,8].

Classical risk factors for venous thromboembolism (VTE)—including surgery, trauma, immobility, malignancy, pregnancy, hormonal therapy, and inherited thrombophilias such as factor V Leiden and prothrombin G20210A—explain only part of the inter-individual variability in thrombotic risk [6,9,10,11,12,13,14]. This has prompted investigation of additional constitutional determinants, including the ABO blood group system [15,16].

Non-O phenotypes (A, B, AB) are consistently associated with higher plasma concentrations of von Willebrand factor (vWF) and factor VIII, generating a procoagulant profile [12,17,18]. Epidemiologic data indicate a 1.5- to 2-fold higher risk of VTE in non-O individuals compared with group O [12,19,20,21]. Large prospective cohorts (e.g., Wolpin 2010; Ohira 2007) confirmed this association for idiopathic and secondary PE [22,23], and several analyses suggest groups A and AB confer the greatest risk [24,25,26,27,28].

Mechanistically, ABO antigens modulate vWF glycosylation and clearance, with group O showing faster turnover and lower steady-state levels [18,29,30,31,32,33]. Elevated vWF promotes platelet adhesion and endothelial dysfunction, both relevant within the pulmonary circulation [8,34,35]. ABO polymorphisms also correlate with inflammatory adhesion molecules (E-selectin, P-selectin), linking coagulation and vascular inflammation [26,36].

Recent evidence, including an overview from the American Society of Hematology (2025), supports a two- to three-fold higher VTE risk among non-O phenotypes [37]. In a large Han Chinese cohort, Sun et al. (2017) found non-O blood types more prevalent in VTE—particularly among younger and unprovoked cases—suggesting age- and context-specific effects [38]. Other reports have examined ABO distribution specifically in PE. Hajizadeh et al. found a higher prevalence of group A and a lower prevalence of group O among Iranian patients with acute PE, without mortality differences [39]. Wang et al. reported that non-O blood groups confer approximately 45% higher PE risk, driven primarily by groups A and AB [40]. Nevertheless, heterogeneity across populations and study designs persists, and evidence regarding recurrence, severity, and outcomes remains inconsistent [8,20,39].

Given the heterogeneity and inconsistency of existing evidence, a systematic review with limited quantitative synthesis was conducted to clarify the relationship between ABO blood group and PE. The objectives were to

1. Assess whether non-O blood groups confer a higher risk of incident PE.
2. Compare risk profiles among specific ABO phenotypes (A, B, AB).
3. Explore associations with PE prognosis, including recurrence, mortality, and CTEPH.

By appraising and summarizing available primary evidence, this work aims to consolidate current knowledge and delineate research gaps regarding the contribution of ABO phenotype to PE risk and outcomes.

## 2. Materials and Methods

### 2.1. Study Design and Reporting Framework

We conducted a systematic review with limited quantitative synthesis of the literature examining the association between ABO blood groups and pulmonary embolism, adhering to principles recommended by PRISMA 2020 [41] (Preferred Reporting Items for Systematic Reviews and Meta-Analyses) (Supplementary Materials).

### 2.2. Information Sources and Search Strategy

We conducted a systematic search of PubMed, Embase, and Web of Science from inception through 1 August 2025, restricted to English-language publications. The search combined MeSH terms and free-text keywords for pulmonary embolism (e.g., “pulmonary embolism,” “PE”) and ABO blood groups (e.g., “ABO,” “blood group,” “blood type”), along with terms for risk, incidence, recurrence, mortality, and severity.

The following PubMed search string was applied (MeSH + free text): ((“Pulmonary Embolism”[Mesh] OR “pulmonary embolism”[tiab] OR “acute pulmonary embolism”[tiab])AND (“ABO Blood-Group System”[Mesh] OR ABO[tiab] OR “blood group”[tiab] OR “blood type”[tiab] OR “blood grouping”[tiab] OR “ABO polymorphism”[tiab] OR “ABO locus”[tiab])AND (risk[tiab] OR incidence[tiab] OR recurrence[tiab] OR recurrent[tiab] OR mortality[tiab] OR severity[tiab] OR outcome*[tiab] OR prognosis[tiab] OR complication*[tiab])AND (cohort[tiab] OR “case–control”[tiab] OR “case–control”[tiab] OR prospectivee[tiab] OR retrospectivee[tiab] OR registry[tiab] OR observational[tiab]))NOT (Review[pt] OR Meta-Analysis[pt] OR systematic[sb]).

The following Embase (EMTREE) search strings were used: (‘pulmonary embolism’/exp OR ‘pulmonary embolism’:ti,ab OR ‘acute pulmonary embolism’:ti,ab) AND (‘abo blood group’/exp OR abo:ti,ab OR ‘blood group’:ti,ab OR ‘blood type’:ti,ab OR ‘blood grouping’:ti,ab OR ‘abo polymorphism’:ti,ab OR ‘abo locus’:ti,ab) AND (risk:ti,ab OR incidence:ti,ab OR recurrence:ti,ab OR recurrent:ti,ab OR mortality:ti,ab OR severity:ti,ab OR outcome*:ti,ab OR prognosis:ti,ab OR complication*:ti,ab) AND (cohort:ti,ab OR ‘case–control’:ti,ab OR ‘case–control’:ti,ab OR prospective:ti,ab OR retrospective:ti,ab OR registry:ti,ab OR observational:ti,ab) NOT (‘systematic review’/exp OR ‘meta-analysis’/exp OR review:pt OR ‘conference abstract’/it).

The following Web of Science (Topic search) search strings were used: TS = (“pulmonary embolism” OR “acute pulmonary embolism”) AND TS = (ABO OR “blood group” OR “blood type” OR “blood grouping” OR “ABO polymorphism” OR “ABO locus”) AND TS = (risk OR incidence OR recurrence OR recurrent OR mortality OR severity OR outcome* OR prognosis OR complication*) AND TS = (cohort OR “case–control” OR “case–control” OR prospective OR retrospective OR registry OR observational) NOT TS = (“systematic review” OR “meta-analysis” OR “review” OR “conference abstract”).

Reference lists of retrieved articles were also screened for additional eligible studies. The review focused on incident and recurrent PE as primary outcomes, with secondary outcomes including short- and long-term mortality, severity markers, and PE-related complications. Studies were included if they reported ABO blood group (phenotype or genotype) and objectively confirmed PE outcomes in adults (≥18 years). Cohort, case–control, and registry-based studies were eligible. Pediatric-only studies, case reports, small case series, conference abstracts without full text, non-human studies, and reviews were excluded. All included studies were screened independently by two reviewers using a predefined eligibility checklist, with discrepancies resolved through discussion.

### 2.3. Eligibility Criteria (PICO Framework)

• Population: Adults (≥18 years), any ancestry.
• Exposure: ABO blood group phenotype or ABO locus genotype.
• Comparator: O vs. non-O, or individual subgroups (A, B, AB vs. O).
• Outcomes: Incident PE and recurrent PE (primary); secondary: short-term mortality (in-hospital/30 days), long-term mortality (≥90 days/1 year), severity markers (hemodynamic instability, right ventricular (RV) dysfunction, Pulmonary Embolism Severity Index (PESI/sPESI), and PE complications (CTEPH).
• Study types: Cohort, case–control, registry-based analyses.
• Excluded: Pediatric-only studies, case reports, small case series, conference abstracts without full text, non-human studies, meta-analyses, systematic reviews, and narrative reviews.
• Timeframe: All years up to 1 August 2025.
• Language: English.

### 2.4. Variables and Definitions

Ancestry is a critical factor to consider in studies of pulmonary embolism because both genetic predisposition and clinical outcomes vary substantially across populations. The distribution of ABO blood groups, factor V Leiden, and other thrombophilic mutations differs by ancestral background, which in turn influences baseline risk [42,43,44]. Epidemiological studies also demonstrate that pulmonary embolism incidence and prognosis vary between individuals of European, African, and Asian ancestry [45,46]. Additionally, pharmacogenetic differences affecting anticoagulant metabolism further highlight the need to account for ancestry when interpreting results [47].

The PESI is a validated risk stratification tool that estimates 30-day mortality in acute pulmonary embolism by combining demographic data, comorbidities, and clinical parameters into a five-class score (I–V) [48]. To improve usability in daily practice, the simplified PESI (sPESI) was developed, which uses six binary variables (age > 80 years, cancer, chronic cardiopulmonary disease, tachycardia, hypotension, hypoxemia). A score of 0 identifies low-risk patients suitable for early discharge or outpatient management, while any score ≥ 1 indicates higher risk [49].

### 2.5. Data Extraction

Data were extracted independently by two reviewers using a standardized template capturing: study characteristics (author, year, design, sample size, and population features), definition of PE, ABO exposure, effect measures (odds ratio [OR], hazard ratio [HR], relative risk [RR]), covariate adjustments, outcomes (mortality, recurrence, severity, complications), and reported limitations.

Effect measures were recorded exactly as presented in the primary studies to preserve methodological fidelity. We did not harmonize effect estimates (e.g., converting HRs to ORs) because the included studies varied in design and outcome ascertainment; retaining the original effect measure preserved methodological fidelity and avoided introducing additional assumptions.

Risk of bias in observational studies was evaluated with the Newcastle–Ottawa Scale (NOS), addressing selection, comparability, and outcome domains. Disagreements in data extraction or risk-of-bias assessment were resolved by consensus. Extraction was performed independently and verified for accuracy.

### 2.6. Data Synthesis

Given methodological heterogeneity, results were primarily summarized descriptively. A formal meta-analysis was conducted only when studies provided directly comparable estimates for PE incidence in non-O versus O groups. Log-transformed ORs and HRs with 95% confidence intervals (CIs) were pooled under a random-effects model (DerSimonian–Laird method) using inverse-variance weighting. HRs and ORs were treated as approximately equivalent, given the low absolute incidence of PE. Between-study heterogeneity was evaluated using the Cochran Q test and quantified with the I^2^ statistic.

Potential publication bias was assessed visually through funnel-plot inspection and, when at least three studies were available, statistically with Egger’s regression; owing to the limited number of studies, these results were interpreted with caution.

All analyses and forest plots were generated within an integrated Python (3.14.0) environment employing the matplotlib and statsmodels libraries.

## 3. Results

### 3.1. Study Selection

**Figure 1:**
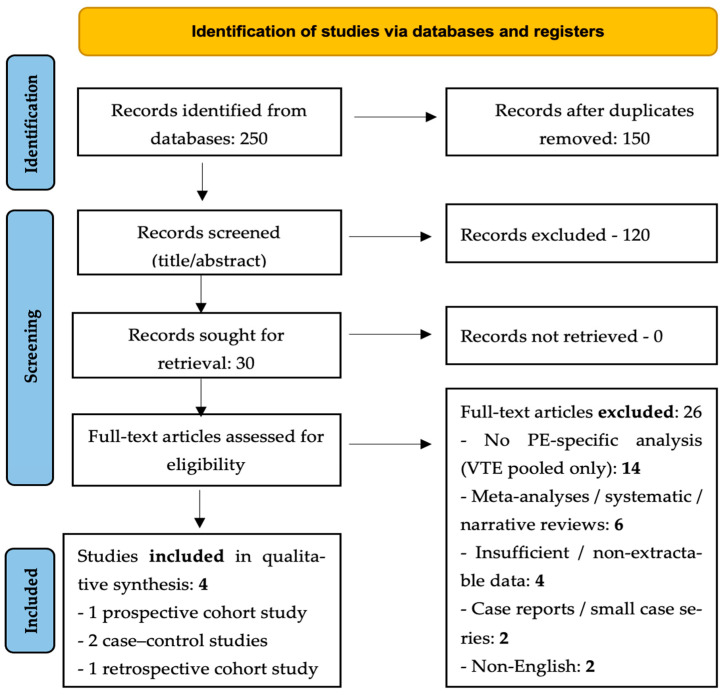
Flowchart of the selection process.

Database searches identified 250 records. After duplicates were removed, 150 unique titles and abstracts were screened. Of these, 120 were excluded as irrelevant or not focused on pulmonary embolism (PE). Thirty full-text articles were assessed; 26 were excluded because PE outcomes were not analyzed separately from venous thromboembolism (*n* = 14), publication type was ineligible (reviews or meta-analyses, *n* = 6), data were non-extractable (*n* = 4), or the reports were case series/case reports (*n* = 2). Two non-English studies were also excluded. Four primary studies fulfilled all inclusion criteria: two prospective cohorts, one retrospective cohort, and one case–control investigation conducted in North America, Europe, and Asia (2010–2021). All provided objectively confirmed PE outcomes and ABO blood-group data. The selection process is illustrated in Figure 1.

### 3.2. Study Characteristics

The four included studies consisted of one prospective cohort, two retrospective cohorts, and one case–control study, conducted in the United States, Iran, China, and Croatia between 2010 and 2021. Sample sizes ranged from 230 to >200,000 participants. PE diagnosis was confirmed mainly by computed tomography pulmonary angiography or ventilation–perfusion scanning.

Wolpin et al. (2010, USA) [22] analyzed data from two prospective population-based cohorts (Nurses’ Health Study and Health Professionals Follow-up Study), following more than 100,000 participants over approximately one million person-years, with 499 objectively confirmed incident PE cases. ABO blood group was assessed by phenotype. The primary comparator was group O versus non-O, with additional subgroup analyses including A, B, and AB groups. Outcomes were limited to incident PE, without evaluation of recurrence, mortality, or severity indices [22].

Hajizadeh et al. (2016, Iran) [39] performed a case–control study including 230 patients with acute PE compared to hospital staff and blood donor controls. ABO phenotype was determined, and comparisons included O versus non-O and individual subgroups A, B, and AB. In addition to analyzing PE incidence, this study uniquely reported in-hospital and follow-up mortality. Severity measures or recurrence were not investigated [39].

Sun et al. (2017, China) [38] reported a large retrospective hospital-based cohort of more than 200,000 inpatients. From this cohort, 1412 venous thromboembolism cases were identified, including 441 with isolated PE and 371 with combined PE and deep vein thrombosis (DVT). ABO phenotype was assessed, with comparisons made between O and non-O groups. Subgroup analyses distinguished PE-only from combined PE + DVT cases. Outcomes were limited to incident PE; recurrence, mortality, and severity indices were not analyzed [38].

Kereš et al. (2021, Croatia) [43] carried out a cross-sectional study including 74 patients with objectively confirmed PE and 303 healthy blood-donor controls. Unlike other studies, this investigation assessed ABO genotype rather than phenotype. Comparisons included O1O1 versus non-O1O1 genotypes and subgroup analyses of A1B, BB, and other allelic combinations. In addition to incident PE, this study evaluated severity using the PESI score. It did not assess recurrence, mortality, or long-term outcomes [43].

Together, the studies provided evidence on the relationship between ABO blood group and pulmonary embolism across >300,000 individuals. Three studies used phenotyping and one genotyping. O versus non-O was the main comparator; A, B, and AB sub-groups were variably analyzed. The primary outcome was incident PE; secondary endpoints (recurrence, mortality, severity) were inconsistently reported. No study assessed CTEPH. Summary characteristics appear in Table 1.

### 3.3. Association Between ABO Blood Group and Pulmonary Embolism

**Figure 2:**
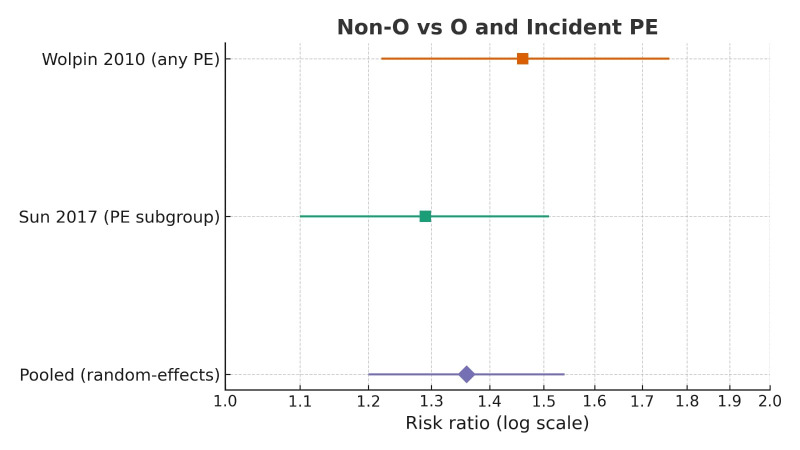
Forest plot of studies assessing risk of pulmonary embolism in non-O vs. O blood groups [22,38]. Squares represent study-specific effect estimates; horizontal lines represent 95% CIs; diamond represents pooled random-effects estimate.

All studies showed a positive association between non-O blood groups and pulmonary embolism (PE) risk (Figure 2).

In the U.S. cohorts analyzed by Wolpin et al. (2010) [22], more than 100,000 participants were followed for approximately one million person-years, identifying 499 incident PE cases. Non-O blood groups were associated with higher risk of idiopathic PE (HR 1.86, 95% CI 1.35–2.57) and any PE (HR 1.46, 95% CI 1.22–1.76); risk estimates were similar across A, B, and AB groups [22].

In the Iranian case–control study by Hajizadeh et al. (2016) [39], 230 patients with acute PE between 2012 and 2014 were compared with hospital controls. Blood group A was significantly more frequent among PE patients compared with controls (46.1% vs.~36%; *p* = 0.002–0.03), while group O was less frequent (25.2% vs. ~37%; *p* = 0.009–0.04). When patients were stratified as O vs. non-O, non-O groups were significantly overrepresented among PE cases. Although in-hospital adverse events and mid-term mortality did not differ significantly between groups, non-O patients exhibited higher overall mortality (*p* = 0.01) [39].

In the Croatian genetic case–control study by Kereš et al. (2021) [43], 74 PE patients and 303 controls were analyzed. Non-O phenotypes, particularly AB, were more frequent among cases (OR 3.9, 95% CI 1.63–9.79). Genotype analysis showed substantially higher risk for BB (OR 11.6, 95% CI 2.00–67.5) and A1B (OR 4.9, 95% CI 1.95–12.42) carriers. PESI-based severity did not differ across ABO groups [43].

Sun et al. (2017) [38] analyzed 200 660 hospitalized Han Chinese patients, identifying 1412 VTE events (441 isolated PE). Non-O groups were more frequent among VTE patients (OR 1.36, 95% CI 1.21–1.54) and remained significant after adjusting for age and sex. For PE alone, the association persisted (OR 1.29); the strongest effects occurred in unprovoked PE (OR 1.86) and patients <39 years (OR 1.99) [38].

### 3.4. Pooled Evidence

Across the four included studies, effect estimates for non-O versus O blood groups ranged from 1.29 to 1.86 in large population-based cohorts and from 3.9 up to 11.6 in genotype-based analyses [22,38,39,43]. Despite differences in study design, population, and methods of blood-group determination, the direction of association was consistent.

The two large population-based studies, Wolpin et al. (2010) [22] and Sun et al. (2017) [38], provided directly comparable estimates suitable for quantitative synthesis. In Wolpin et al., non-O individuals had a higher risk of PE (HR 1.46, 95% CI 1.22–1.76); in Sun et al., the corresponding OR was 1.29 (95% CI 1.10–1.51).

**Figure 3:**
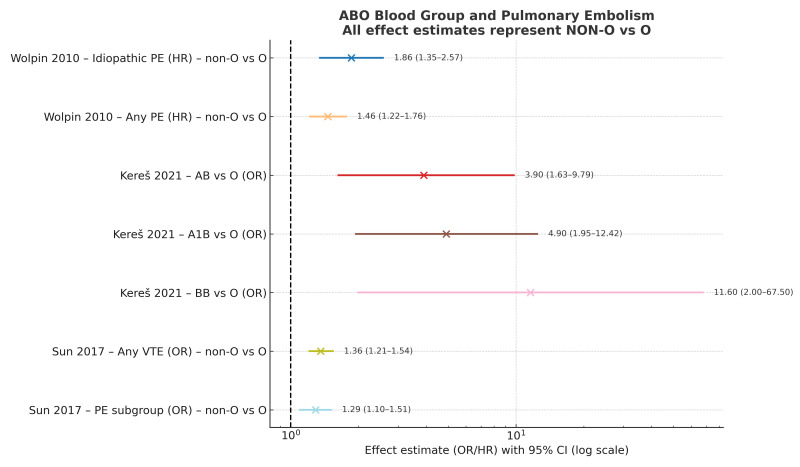
Forest plot showing the association between ABO blood groups and pulmonary embolism [22,38,39,43]. Non-O blood types were consistently associated with increased risk compared with group O.

A random-effects meta-analysis (DerSimonian–Laird method, inverse-variance weighting) yielded a pooled relative risk of 1.36 (95% CI 1.20–1.54; I^2^ = 2.6%), indicating an approximately 36% higher risk of incident PE among non-O blood groups (Figure 3). Between-study heterogeneity was minimal.

Other studies contributed valuable descriptive evidence, but could not be quantitatively pooled. Hajizadeh et al. (2016) [39] reported a higher frequency of group A and a lower frequency of group O among Iranian patients with acute PE, though without reporting adjusted effect estimates. Kereš et al. (2021) [43] analyzed genotypes, identifying markedly elevated risk in carriers of A1B and BB alleles, but these results were not directly comparable to phenotype-based O versus non-O analyses.

### 3.5. Publication Bias Assessment

**Figure 4:**
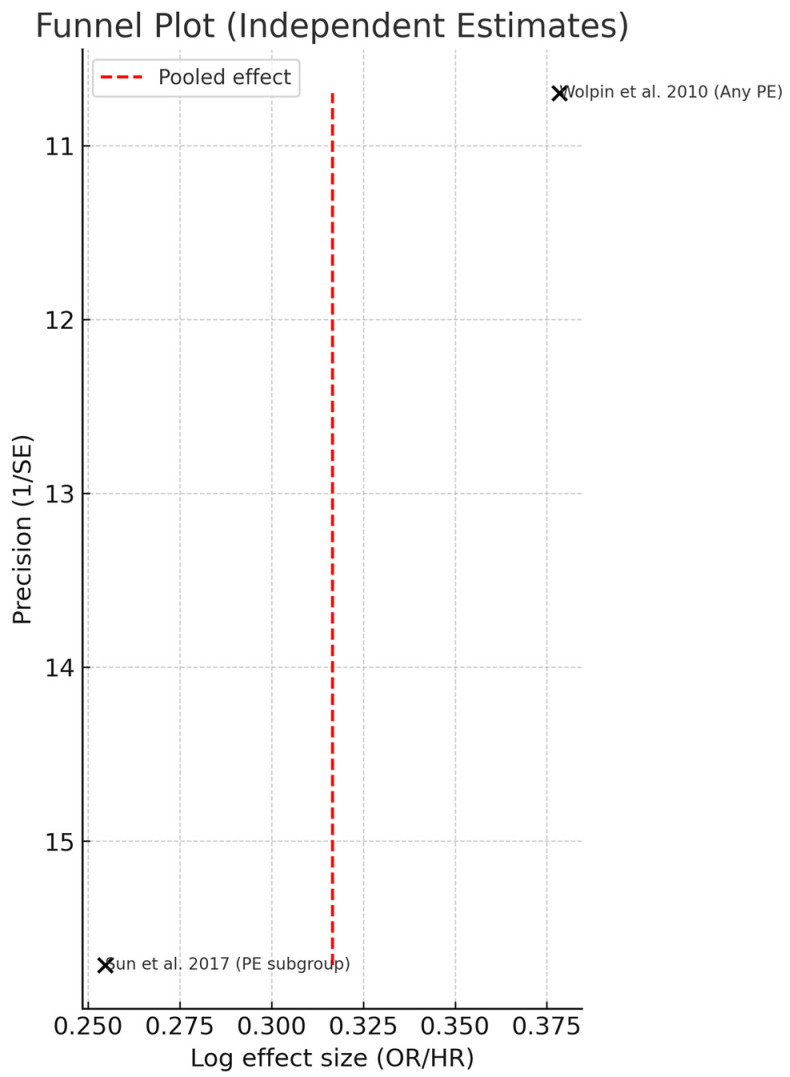
Funnel plot assessing publication bias in studies evaluating the association between ABO blood group and pulmonary embolism (independent estimates) [22,38]. The red line marks the marks the pooled log-effect estimate.

Publication bias was evaluated using a funnel plot and Egger’s regression test, restricted to studies providing directly comparable adjusted estimates for the association between non-O versus O blood groups and incident pulmonary embolism (Wolpin et al., 2010 [22]; Sun et al., 2017 [38]). The remaining studies were excluded from this analysis because of differences in exposure definition, outcome specification, or lack of adjusted quantitative estimates. Hajizadeh et al. reported only unadjusted frequencies without standardized effect sizes, while Kereš et al. evaluated genotypes rather than phenotypic blood groups and analyzed distinct allelic contrasts (A1B, BB vs. O1O1). Including these heterogeneous data would have produced non-comparable effect estimates. Visual inspection of the funnel plot suggested mild asymmetry (Figure 4). The red line marks the pooled log-effect estimate, serving as the reference around which individual study estimates would be expected to distribute symmetrically. Because only two studies contributed and directly comparable estimates, the line simply indicates the pooled effect rather than functioning as a regression line. However, this finding should be regarded as inconclusive. Egger’s regression was not statistically significant (*p* > 0.1).

## 4. Discussion

The available evidence suggests that non-O blood groups are associated with a modestly increased risk of pulmonary embolism (PE). Across four observational studies from North America, Europe, and Asia, the direction of effect was consistent: individuals with non-O phenotypes had higher risk of incident PE than those with blood group O. Reported effect estimates ranged from 1.29 to 1.86 in large population cohorts and were higher in genotype-based analyses [22,38,39,43].

The strongest evidence came from the prospective U.S. cohorts of Wolpin et al. (2010) [22], which demonstrated a nearly two-fold increase in idiopathic PE risk among non-O individuals after multivariable adjustment. This pattern was uniform across A, B, and AB subgroups, implying a protective effect of the O phenotype [22]. The large Chinese cohort by Sun et al. (2017) corroborated these findings, showing elevated risks across all non-O groups, particularly in younger and unprovoked cases, consistent with an independent contribution of ABO status to thrombotic susceptibility [38].

Smaller hospital-based studies provided complementary data. Hajizadeh et al. (2016) observed a higher frequency of blood group A and lower frequency of O among acute-PE patients, with a trend toward higher mortality in non-O groups [39]. Kereš et al. (2021) extended this to the genetic level, identifying A1B and BB genotypes as strong risk markers (OR 4.9 and 11.6, respectively), although sample size was limited [43]. These observations reinforce biological plausibility given the established relationship between ABO polymorphisms, von Willebrand factor, and factor VIII concentrations [50,51].

When synthesized, the available data indicate that ABO blood group represents a biologically relevant, though modest, determinant of thrombotic risk. The pooled analysis showed an approximately 36% increase in PE risk among non-O individuals with minimal heterogeneity, supporting consistency, but not direct causality, across diverse populations, study designs, and ascertainment methods, but not direct causality. Evidence regarding severity, recurrence, or mortality remains insufficient [12,19,21,52,53].

Several limitations must be acknowledged. The number of eligible studies was small, restricting subgroup analyses and statistical power. Heterogeneity in study design, ABO ascertainment (self-reported, serologic, or genotypic), and adjustment for confounders introduces potential bias. Only one study evaluated mortality and severity outcomes, and residual confounding cannot be excluded. Furthermore, differences in ancestry, which influence both ABO distribution and thrombophilic mutations, may limit generalizability.

Despite these constraints, the findings have potential clinical relevance. ABO blood group is routinely available, immutable, and low-cost, and could complement existing risk stratification models, especially for unprovoked or recurrent events [54,55]. While ABO status alone cannot guide prophylaxis, it may refine individualized assessment in younger patients or those lacking classical risk factors [8,13,40]. Integration of ABO phenotype or high-risk genotypes with established thrombophilia markers could improve risk prediction and inform future research on personalized anticoagulation strategies [25,46,47,56,57,58].

## 5. Conclusions

### 5.1. Summary of Findings

This systematic review and limited quantitative synthesis indicates that non-O blood groups are associated with a modest but consistent increase in pulmonary embolism risk across diverse populations. The pooled data suggest an approximately 30–40% elevation in risk among non-O individuals, with low statistical heterogeneity and convergent findings across study designs. Evidence regarding recurrence, severity, and mortality remains sparse and insufficient for definitive conclusions.

### 5.2. Clinical and Research Implications

Although ABO status is not a determinant for clinical decision-making on its own, its routine availability and biological plausibility support its potential role as a supplementary risk marker within multifactorial prediction models. Future large-scale, ancestry-diverse cohorts, with standardized outcome definitions and genotype–phenotype analyses are needed to clarify causal mechanisms, quantify genotype-specific risks, and determine whether ABO information can meaningfully enhance thromboembolic risk stratification.

## Figures and Tables

**Table 1:** Characteristics of included studies on ABO blood groups and pulmonary embolism.

Author (Year, Country)	Design/Population	Sample Size (PE Cases/Controls or Cohort)	Exposure	Comparators	Outcomes Assessed
**Wolpin (2010, USA)** **[22]**	Prospective cohort; Nurses’ Health Study and Health Professionals Follow-up Study, general U.S. population	>100,000 participants followed over~1 million person-years; 499 incident PE	Phenotype	O vs. non-O as primary; subgroup analyses of A, B, and AB	Incident PE identified prospectively during follow-up
**Hajizadeh (2016, Iran)** **[39]**	Case–control; hospitalized PE patients vs. blood donors and staff controls	230 PE/230 controls	Phenotype	O vs. non-O; subgroup comparisons A, B, AB	Incident acute PE; short-term (in-hospital) mortality; follow-up mortality
**Sun (2017, China)** **[38]**	Retrospective hospital-based cohort; large inpatient population	>200,000 inpatients screened; 1412 VTE cases (441 isolated PE; 371 PE + DVT)	Phenotype	O vs. non-O; subgroup analyses separating PE-only from combined PE + DVT	Incident PE identified within hospitalization; subgroup analysis by isolated PE vs. combined PE + DVT
**Kereš (2021, Croatia)** **[43]**	Case–control; confirmed PE patients vs. healthy controls	74 PE/303 controls	Genotype	O1O1 vs. non-O1O1 genotypes; subgroup analyses of A1B, BB, and other allele combinations	Incident PE; severity assessed by PESI

## Data Availability

The data presented in this study are available PubMed, Embase and Web of Science. These data were derived from publicly accessible sources: National Institutes of Health (NIH)—available at https://pubmed.ncbi.nlm.nih.gov (accessed on 20 August 2025); Excerpta Medica dataBASE—available at https://www.embase.com/landing?status=grey (accessed on 18 August 2025); Web of Science (WoS)—available at https://access.clarivate.com/ (accessed on 20 August 2025). All data are from public domain sources and are freely available.

## Supplementary Materials

The following supporting information can be downloaded at: https://www.mdpi.com/article/10.3390/diagnostics15232973/s1, PRISMA 2020 Checklist. Reference [41] is cited in the supplementary materials.

## Abbreviations

The following abbreviations are used in this manuscript:

CTEPH	Chronic Thromboembolic Pulmonary Hypertension
CTPA	Computed Tomography Pulmonary Angiography
DVTDeep	Vein Thrombosis
PE	Pulmonary Embolism
PESI	Pulmonary Embolism Severity Index
sPESI	Simplified Pulmonary Embolism Severity Index
VTE	Venous Thromboembolism
vWF	von Willebrand Factor
